# A Genome-Wide Association Study of Idiopathic Dilated Cardiomyopathy in African Americans

**DOI:** 10.3390/jpm8010011

**Published:** 2018-02-26

**Authors:** Huichun Xu, Gerald W. Dorn II, Amol Shetty, Ankita Parihar, Tushar Dave, Shawn W. Robinson, Stephen S. Gottlieb, Mark P. Donahue, Gordon F. Tomaselli, William E. Kraus, Braxton D. Mitchell, Stephen B. Liggett

**Affiliations:** 1 Division of Endocrinology, Diabetes and Nutrition, Department of Medicine, University of Maryland School of Medicine, Baltimore, MD 21201, USA; parihar1.ankita@gmail.com (A.P.); tushardave26@gmail.com (T.D.); bmitchel@som.umaryland.edu (B.D.M.); 2 Center for Pharmacogenomics, Department of Internal Medicine, Washington University School of Medicine, St. Louis, MO 63110, USA; gdorn@dom.wustl.edu; 3 Institute for Genome Sciences, University of Maryland School of Medicine, Baltimore, MD 21201, USA; AShetty@som.umaryland.edu; 4 Division of Cardiovascular Medicine, University of Maryland School of Medicine, Baltimore, MD 21201, USA; Srobinso@som.umaryland.edu (S.W.R.); Sgottlie@som.umaryland.edu (S.S.G.); 5 Division of Cardiology, Department of Medicine, Duke University Medical Center, Durham, NC 27708, USA; mark.donahue@duke.edu (M.P.D.); william.kraus@duke.edu (W.E.K.); 6 Department of Medicine, Division of Cardiology, Johns Hopkins University, Baltimore, MD 21218, USA; gtomasel@jhmi.edu; 7 Duke Molecular Physiology Institute, Duke University Medical Center, Durham, NC 27701, USA; 8 Geriatrics Research and Education Clinical Center, Baltimore Veterans Administration Medical Center, Baltimore, MD 21201, USA; 9 Department of Internal Medicine and Molecular Pharmacology and Physiology, and the Center for Personalized Medicine and Genomics, University of South Florida Morsani College of Medicine, Tampa, FL 33612, USA; For the Genetics of African American Heart Failure (GAAHF) Consortium

**Keywords:** idiopathic dilated cardiomyopathy, GWAS, CACNB4, calcium channel, African American, heart failure

## Abstract

Idiopathic dilated cardiomyopathy (IDC) is the most common form of non-ischemic chronic heart failure. Despite the higher prevalence of IDC in African Americans, the genetics of IDC have been relatively understudied in this ethnic group. We performed a genome-wide association study to identify susceptibility genes for IDC in African Americans recruited from five sites in the U.S. (662 unrelated cases and 1167 controls). The heritability of IDC was calculated to be 33% (95% confidence interval: 19–47%; *p* = 6.4 × 10^−7^). We detected association of a variant in a novel intronic locus in the *CACNB4* gene meeting genome-wide levels of significance (*p* = 4.1 × 10^−8^). The *CACNB4* gene encodes a calcium channel subunit expressed in the heart that is important for cardiac muscle contraction. This variant has not previously been associated with IDC in any racial group. Pathway analysis, based on the 1000 genes most strongly associated with IDC, showed an enrichment for genes related to calcium signaling, growth factor signaling, neuronal/neuromuscular signaling, and various types of cellular level signaling, including gap junction and cAMP signaling. Our results suggest a novel locus for IDC in African Americans and provide additional insights into the genetic architecture and etiology.

## 1. Introduction

Annually, an estimated 915,000 new cases of heart failure are diagnosed, and 65,000 deaths are attributed to the syndrome [1]. Nearly half of these cases are not due to ischemic heart disease [2]. Of these non-ischemic cardiomyopathies, idiopathic dilated cardiomyopathy (IDC) is one of the most well characterized clinical entities and is the subject of the current work.

At least 30% of patients with IDC have an affected family member [3,4,5]. However, the etiology of a significant number of IDC cases has yet to be defined, even after extensive genetic screening. Varying clinical features of cardiomyopathy support a complex etiology even when monogenic mutations are involved. For example, it is known that the same clinical phenotype can be caused by different gene mutations, and conversely, mutations in the same gene can cause different clinical subsets of cardiomyopathy in different individuals [6]. These observations suggest that genetic, environmental, or other non-genetic modifiers could interact with myopathic-predisposing mutations to influence the risk of development and prognosis.

A notable feature of IDC is its higher prevalence in African Americans compared as to European Caucasians (relative odds = 2.6 with 95% confidence interval (CI) 1.6–4.3) [7,8]. For example, African American children (<18 years old) have a higher annual incidence of dilated cardiomyopathy than Caucasian children (0.98 versus 0.46 cases per 100,000) [9]. Whether African Americans with IDC also experience worse outcomes compared to European Caucasians has not been adequately studied [10], although numerous studies have reported increased mortality from heart failure in African Americans [11,12,13]. 

It has been reported that African American athletes exhibit a greater magnitude of left ventricular hypertrophy (LVH) in response to intense physical exercise than white athletes, suggesting potential biological differences in cardiac adaptation between African Americans and Caucasians [14]. However, despite the higher burden of IDC in African Americans, the three genome wide association studies (GWASs) of primary cardiomyopathy currently reported in the National Human Genome Research Institute (NHGRI) GWAS catalog (2 studies on IDC, 1 study on hypertrophic cardiomyopathy) are all based on populations with European ancestry [15]. In contrast, our current study is based exclusively in subjects with self-reported African American ancestry. Focusing on African Americans offers two potential advantages. First, the distinctive genetic architecture of populations with African ancestry may provide opportunities to fine map causative genetic variants and to generalize findings obtained from European GWASs [16]. Second, studying IDC in African Americans provides potential opportunities to understand the ethnic differences in this disease and its progression, which may provide insights into prevention and treatment strategies. Here we report the findings from the first African American IDC GWAS, identifying a novel variant in a calcium voltage-gated channel with genome-wide significance, and pathway enrichment of a network of proteins with near GWAS-level significance both known and not known to be related to cardiomyopathy, consistent with a unique African risk profile for IDC.

## 2. Materials and Methods

### 2.1. Subjects

The Genetics of African American Heart Failure (GAAHF) consortium recruited 775 IDC cases from five sites in the U.S. (Supplementary Table S1). Local institutional review board (IRBs) from individual sites approved this study protocol, and informed consent was obtained from all participants. All subjects self-identified as being African American and ranged in age from 26 to 85 years. Subjects received a medical history interview, physical examination, routine laboratory tests, electrocardiogram, and echocardiogram. In some cases, coronary angiogram, a computed tomography (CT), or magnetic resonance imaging (MRI) was performed when cardiac ischemia was suspected. All subjects had ejection fractions (EF) ≤ 40%, left ventricular end diastolic dimensions (LVEDD) ≥ 5.6 cm, and New York Heart Association (NYHA) class III–IV symptoms. Ninety percent of patients were receiving β-blockers and/or angiotensin-converting enzyme inhibitors or angiotensin receptor antagonists. Those with a family member within three generations having medical history of heart failure were considered to have familial cardiomyopathy and were excluded.

As controls for this study, we utilized 1167 self-identified African American participants from the Multiethnic Study of Atherosclerosis (MESA), who had previously undergone genome-wide genotyping with the same array (i.e., the Affymetrix Genome-Wide Human SNP Assay *6.0* from Affymetrix, Inc., based in Santa Clara, CA, USA) at the Affymetrix Research Services Laboratory and genotype calling based on the Affymetrix Birdseed software. MESA data were obtained from the database of Genotypes and Phenotypes (dbGAP) (Accession: phs000209.v13.p3). Using publicly available controls is an efficient and robust strategy for conducting GWASs provided that appropriate quality control procedures are followed to ensure comparability of genetic data between cases and the public controls and the control of potential population structure differences between case and public controls [17,18]. We and others have successfully used this strategy [19,20,21]. MESA, conducted across six sites in USA, is a population-based study of cardiovascular disease in asymptomatic men and women aged 45–84 years old without known cardiovascular diseases, including heart failure, at enrollment. 

### 2.2. Genotyping and Data Quality Control Analysis

DNA from cardiomyopathy cases was genotyped in the Genomics Core of the University of Maryland using the Affymetrix Genome-Wide Human SNP Assay *6.0* (1 million SNPs). Genotypes were called using the Affymetrix Birdseed software. 

After excluding monomorphic SNPs, there were 908,343 SNPs genotyped among cases and 909,622 SNPs genotyped among controls. Quality control and filtering were performed at the sample and SNP level (workflow chart: Supplementary Figure S1). Samples were excluded if they had high missing genotyping call rates (>1.5%), excessive heterozygosity, discordant sex determination between genotyped data and phenotypic data, or relatedness with other study subjects identified by the Identity-by-descent calculation in PLINK (Pi-hat > 0.125 for relatedness). A total of 662 cases and 1138 controls were retained for analysis.

For genotype level quality control, SNPs were excluded for any of the following: high missing call rate (>1%), differential missingness between cases and controls (*p* < 1 × 10^−3^), or HWE *p* < 1 × 10^−3^ in controls, duplication, or ambiguous genomic annotation. Our analysis focused on autosomes only. These filtering criteria resulted in the retention of 685,400 SNPs for an initial GWAS with only directly genotyped data. Based on this initial GWAS, we additionally excluded any SNP that showed evidence for association at *p* < 1 × 10^−4^ but for which there was no evidence of association (*p* < 1 × 10^−3^) for SNPs in high LD (*R*^2^ > 0.5) with the index SNP. We further compared minor allele frequency (MAF) differences between our IDC samples and two existing African American genome-wide genotype datasets: (1) the non-stroke samples from the Genetics of Early Onset Stroke (GEOS) study [22]; and (2) the Hapmap ASW samples. SNPs were excluded if the difference in MAF was >10%.

### 2.3. Principal Component Analysis

Principal component (PC) analysis was conducted using EIGENSOFT (5.0.2) [23] to account for population structure differences between case and control samples after LD pruning (window size 100 bp, sliding window size of 5 bp, and LD threshold *r*^2^ = 0.2) (Supplementary Figure S2). The genetic variance accounted for by each PC is plotted in Supplementary Figure S3. Logistic regression analysis, controlling for age and gender, showed that PC1, PC9, and PC10 were significantly correlated with disease status (*p* < 5.0 × 10^−2^). Therefore, the first 10 PCs were included as covariates for final GWA analysis. We also merged our genotype data with the Hapmap 3 reference genome data and performed PC analysis to verify the genetic ancestry of our samples. The results are consistent with self-reported African American ancestry for our samples. As expected, the genetic ancestry profiles of our samples lie between European Caucasian (CEU) and Yoruba from West Africa (YRI) (Supplementary Figure S4).

### 2.4. Heritability Analysis

The heritability of IDC was estimated using the Genome-wide Complex Trait Analysis (GCTA) tool [24]. This approach defines heritability as the proportion of phenotypic variance explained by the full genome-wide array of SNPs. GCTA estimates the phenotype variance explained by SNPs by fitting all the SNPs simultaneously in the model as random effects in a mixed linear model [25]. Age, gender, and 10 PCs accounting for population structure were included as covariates in these analyses.

### 2.5. Imputation 

Following quality control of the case and control genotypes, genotypes at additional loci were imputed jointly based on the 1000-genome phase 1 reference panel (integrated_phase1_v3.20101123) [26]. Sex chromosomes were excluded before imputation. Imputation was based on 685,386 SNPs. Pre-phasing was conducted in SHAPEIT2 [27] and imputation was performed using IMPUTE2 following standard procedure [28]. Imputation resulted in generation of 27,494,467 SNPs. The imputation quality was assessed by the imputation quality score “info” for imputed SNPs. An info score of one indicates that there is no uncertainty for the imputed genotypes, while an info score of zero indicates a complete guess solely based on allele frequency in the population. The quality of imputation was very high with estimated ‘info” scores of 0.9–1 for most imputed SNPs (Supplementary Figure S5). Consistent with standard practices, we excluded imputed SNPs with info scores <0.30. As a second metric of imputation quality, we estimated concordance between imputed-back genotypes and original genotypes for directly genotyped SNPs. Also reflecting high quality imputation, we observed a concordance of 90–100% for most directly genotyped SNPs (Supplementary Figure S5).

### 2.6. GWAS Association Analysis and Post GWAS Quality Control

The genetic association analyses were carried out under an additive genetic model using logistic regression with the SNPTEST software [29]. All analyses were adjusted for age, sex, and the first 10 PCs. SNPs were removed from analysis if they had an imputation quality score “info”<0.3, HWE *p* < 1 × 10^−6^, or MAF < 1%. 

### 2.7. Pathway Analysis

We assigned SNPs to nearest genes based on their genomic locations. Intragenic SNPs that were located within more than one gene were not assigned a nearest gene. Intergenic SNPs were assigned to the nearest gene if the distance was less than 40 kb. The top 1000 genes were then subjected to pathway enrichment analysis in two complementary databases: the Kyoto Encyclopedia of Genes and Genomes (KEGG) pathways in the Database for Annotation, Visualization and Integrated Discovery (DAVID) v6.7 [30] and QIAGEN’s Ingenuity® Pathway Analysis (IPA®, QIAGEN Redwood City, www.qiagen.com/ingenuity). 

## 3. Results

### 3.1. Heritability Analysis 

The characteristics of participants are summarized in Supplementary Table S1. Heritability analysis, based on directly genotyped autosomal single nucleotide polymorphisms (SNPs) and assuming 0.04% disease prevalence, revealed that genomic variation accounted for 33% (95% confidence interval 19–47%, *p* = 6.4 × 10^−7^) of susceptibility to IDC after accounting for age, gender, and population structure [24]. Additional analyses revealed that most of the genetic variance in IDC was attributable to markers on chromosomes 1, 2, 3 and 7 (Supplementary Figure S6).

### 3.2. Genome-Wide Single Variant Association Analysis

**Figure 1:**
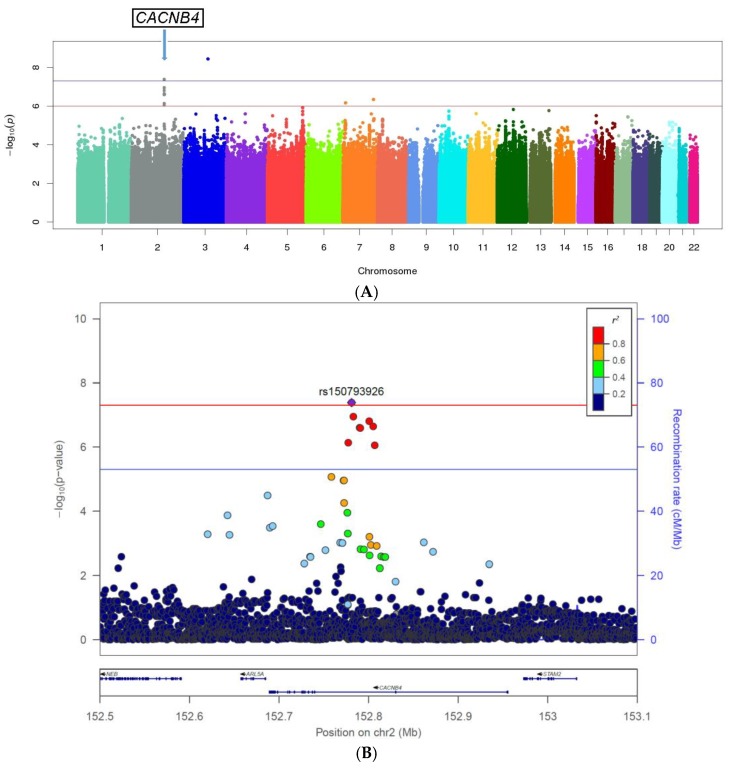
(**A**) Manhattan plots of the genome-wide association results based on imputed data. The *x*-axis shows the chromosome number, while *y*-axis shows the −log (*p*). A post genome-wide association study (GWAS) filter was applied including imputation quality measure “info” >0.3, Hardy–Weinberg equilibrium (HWE) *p* > 1.0 × 10^−6^, minor allele frequencies (MAFs) >1%; (**B**) Regional association plot with linkage disequilibrium (LD) structure for the novel *CACNB4* loci. Genomic coordinates are shown on the *x*-axis, and –log (*p*) is shown on the *y*-axis to the left. The index genetic variant is shown in purple. The *r*^2^ values of the remaining genetic variants with the index variant are color-coded as indicated by the color bar to the upper right. The genes in this region are indicated at the bottom.

The GWAS results, excluding SNPs or short indels with imputation quality measure “info” <0.3, Hardy–Weinberg equilibrium (HWE) *p* < 1.0 × 10^−6^, or minor allele frequencies (MAFs) <1%, are shown as a Manhattan plot in Figure 1A. Eleven SNPs showed an association with *p* < 1.0 × 10^−6^ (Table 1, Supplementary Table S2), including one cluster on chromosome 2q reaching genome-wide statistical significance (peak variant: rs150793926; odds ratio (OR) = 0.49; *p* = 4.1 × 10^-8^). Rs150793926 is an intronic genetic variant in *CACBN4*, a gene encoding a subunit of a voltage-dependent calcium channel (Figure 1B). A SNP at a second locus also achieved genome-wide significance (rs1846594 on chromosome 3), but nearby SNPs in linkage disequilibrium (LD) with rs1846594 did not show supporting associations with IDC; thus, this SNP did not meet our standard for further follow-up. The lambda for the genome-wide association results was 0.99 after post-GWAS quality filtering, showing no evidence for genomic inflation (Supplementary Figure S7). 

To assess whether any additional genetic variants at the *CACNB4* locus were independently associated with cardiomyopathy, we performed a conditional analysis. After including the top variant rs150793926 in this locus as a covariate, no other association signal in this region was observed (Supplementary Figure S8). 

We also tested whether five previously reported GWAS signals that have been associated with IDC in Caucasians were also associated with IDC in African Americans in our study [31,32,33]. There was no evidence for an association with IDC for four of these five SNPs (OR = 0.82–1.44, *p* > 0.01) (Supplementary Table S3); however, we did detect a potential association with the fifth SNP, rs1739843, (*p* = 4.8 × 10^−3^). For this SNP, we observed nearly the same effect size in African Americans as was previously reported in Caucasians [32]: OR for the C allele 1.33 (1.14–1.55, 95% CI) in our study vs. 1.39 (1.54–1.28, 95% CI) previously. Rs1739843 is located in an intronic region of *HSPB7*.

### 3.3. Pathway Analysis Based on Genome-Wide Single Variant Association Analysis

**Figure 2:**
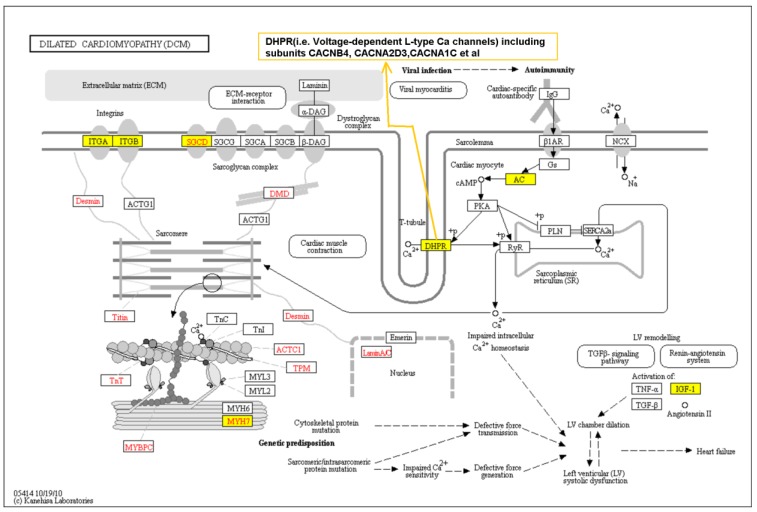
Kyoto Encyclopedia of Genes and Genomes (KEGG) dilated cardiomyopathy pathway overlaid with the top 1000 genes (in yellow box) indicated in our current genome wide association study of idiopathic dilated cardiomyopathy (IDC) in African Americans. Genes with mutations that have been reported in IDC are colored in red font.

To provide a systems view of the genetic contribution to IDC, we performed a pathway analysis of the top 1000 genes tagged by our top 3886 SNPs/indels from the GWAS results. Pathway analysis using the Ingenuity database revealed an enrichment of associations for SNPs located in/near genes with known cardiomyopathy-related pathways such as hypertrophy, nuclear factor of activated T cells (NFAT) cardiac signaling, calcium signaling, gap junction signals, and cAMP signaling being over-represented (False Discovery Rate, i.e. FDR, corrected *p* < 0.1) (Table 2). There was also an enrichment for genes related to neuronal/neuromuscular synaptic signaling. We also performed Kyoto Encyclopedia of Genes and Genomes (KEGG) pathway analysis using the DAVID tool. Consistently, we found that genes known to be related to primary cardiomyopathy, including hypertrophic cardiomyopathy (HCM) and IDC, were significantly enriched (KEGG pathway *p* = 0.004–0.06) in our GWAS signals (Supplementary Table S4). Figure 2 shows the dilated cardiomyopathy pathway from the KEGG pathway database [34] overlaid with genes reported in the literature to have deleterious mutations associated with IDC (depicted in red font) and genes associated with IDC based on GWA analysis of our dataset (depicted in blue font). Note that *MYH7* and *SGCD*, which are modestly, albeit not significantly, associated with IDC in our current GWAS, have mutations reported in IDC cases. The central role of voltage-gated L-type calcium channels in regulating intracellular Ca^2+^ is shown. 

### 3.4. Gene-Based Association Analysis 

We also performed gene-based association analyses using two complementary approaches. First, we performed a set-based aggregation analysis in which we aggregated SNPs based on a gene boundary (±50 kb window) using the fastBAT method, which derives the association p-value for a set of SNPs from an approximated distribution of the sum of chi-squared statistics [35]. Second, we performed an eQTL-based analysis using PrediXcan [36]. PrediXcan predicts the genetically determined transcriptome using an eQTL database specific for cardiac ventricle tissue, and correlates the predicted gene expression with phenotypes. No gene reached genome-wide significance. The most strongly associated gene was *CSPG5* (*p* = 6.72 × 10^−5^) for fastBAT analysis (Supplementary Table S5) and *PRMT7* (*p* = 1.3 × 10^−4^) for PrediXcan analysis (Supplementary Table S6). *CACNB4* was not significantly associated with IDC in either analysis (*p* > 0.05).

## 4. Discussion

To our knowledge, only three GWAS of IDC have been previously published and none included African Americans [31,32,33]. Our analysis indicated that common genetic variations present in African American populations account for 33% of the risk for IDC. We identified a novel intronic locus within *CACNB4* (the voltage-dependent calcium channel beta 4 subunit, also known as the dihydropyridine-sensitive L-type calcium channel β-4 subunit) associated with the risk of IDC with genome-wide significance (*p* = 4.1 × 10^−8^). *CACNB4* is expressed in multiple human tissues including myocytes and neuronal cells (see genecards.org), as well as the developing hearts of zebrafish [37], rodents [38], and canines [39]. L-type calcium channels consist of a complex of α_1_, α_2_/δ, β, and γ subunits at a 1:1:1:1 ratio. While the α_1_ subunit is the main component containing the channel pore, β subunits are critical in trafficking the channels to the plasma membrane, increasing peak calcium current, shifting the voltage dependencies of activation and inactivation, and regulating channel gating through interaction with protein kinases or G proteins [40]. The mammalian β subunit of this calcium channel has four subfamilies (β_1_–β_4_), each encoded by different genes (*CACNB1*–*CACNB4*) with each gene having splicing variants. Thus, *CACNB4* plays an important role in regulating calcium signaling, which in turn may help regulate cardiac muscle contraction. This protein is reported to interact with mutations of sarcomere-related genes in cardiomyopathy according to a canonical KEGG pathway database as shown in Figure 3 [41]. More direct evidence supporting the association of *CACNB4* and other ion-channel genes with IDC comes from gene expression analysis of human ventricular samples from patients with IDC: from 548 ion channel-related genes, 26 genes including *CACNB4* were identified as differentially regulated in the cardiac tissues with IDC [42].

The genome-wide significance of the association between IDC and variation of the L-type calcium channel has additional biological plausibility based on the results of some clinical trials with calcium channel antagonists (blockers), particularly when pooled for meta-analysis [43,44]. In patients treated for hypertension, these studies have shown that those treated with calcium channel blockers had an increased risk of developing heart failure compared to those receiving β-blockers, thiazide diuretics, or angiotensin-converting enzyme inhibitors. It is also noteworthy that some studies have shown an increased antihypertensive response to calcium channel blockers in African Americans compared to Caucasians [45].

There are eight genetic variants in the intronic region of the *CACNB4* gene that we found to be associated with cardiomyopathy with *p* < 1 × 10^−6^. We closely examined this 48.3-Kb region (chr2: 152758611-152806958) using the Encyclopedia of DNA Elements (ENCODE) resource [46]. Interestingly, this region is enriched with histone modification H3k27ac, indicating the presence of active regulatory elements, especially enhancers and various transcription factor binding sites (Supplementary Figure S9). This is consistent with the presence of DNA-sensitive loci in this region (Supplementary Figure S9). It is also interesting that there is a predicted non-coding *Mir-584* gene in this region located near a histone modification H3k27ac peak. *Mir-584* is down-regulated in blood cells in coronary artery disease [47] and is involved in the etiology of hypertension [48]. It is tempting to speculate that the genetic polymorphisms in this region may affect the activities of regulatory elements, either enhancers or transcription factor binding or non-coding microRNA, to regulate gene expression or splicing of *CACNB4* transcripts. There are at least four transcript products of *CACNB4* genes due to alternative splicing. We did not find direct expression quantitative trait locus (eQTL) evidence in the Genotype-Tissue Expression (GTEx) [49] database for these eight tag genetic variants at the *CACNB4* locus, nor with an additional six genetic variants in LD with them (*r*^2^ > 0.2). All 14 of these SNPs are intronic genetic variants within *CACNB4*. Further studies will be needed to delineate the exact mechanisms on how the observed genetic variants regulate cardiomyocyte function.

To evaluate whether our single-variant level correlation analysis results would provide mechanistic insights into the etiology of IDC in African Americans, we examined the enriched pathways in the top GWAS signals. Cardiomyopathy pathways, including those described for IDC, HCM, and arrhythmogenic right ventricular cardiomyopathy (ARVC), and cardiac hypertrophy signals are significantly overrepresented (Table 2 and Supplementary Table S4). At the cellular level, the enriched pathways are indeed recognized to play roles in cardiomyopathy pathogenesis, such as cAMP-related signals [50] and gap junction signals [51]. Consistent with our novel finding on calcium channel subunit *CACNB4* loci, intracellular calcium-related signals are also over-represented. Of note, among the top 1000 genes are six that are known to harbor previously identified mutations associated with rare familial HCM/IDC (*GPD1L*, *MYH6*, *MYH7*, *PSEN2*, *GATAD1*, and *SGCD*). Specifically, *MYH7* and *SGCD* genes have mutations reported in IDC. These six genes are mainly involved in cardiac muscle contraction function. 

Neuronal signals were also significantly enriched in our top GWAS results (Table 2 and Supplementary Table S4). Considering the cardiac muscle function is under direct control of neuromuscular junction, this is not surprising. However, known cardiomyopathy mutations do not include any neuronal genes. Our finding may direct future mechanistic studies of cardiomyopathy in new avenues, including pathways not yet implicated in IDC pathogenesis. We also replicated in this African American sample an association with one (of four) SNPs previously associated with IDC in European Caucasians, located in the intronic region of *HSPB7*.

There are several limitations to our study. The major limitation is that there are no other large collections of African American IDC cases available for replication of the rs150793926 association, at least none of which we are aware. The best variant rs150793926 in *CACNB4*, with genome-wide significance, and the other seven variants in this locus with *p* < 1 × 10^−6^ were not present in earlier GWAS data of IDC in European Caucasians. 

In summary, our study suggests that, in addition to rare exonic mutations, common genetic polymorphisms can also explain a significant portion of the etiology of IDC. Our results suggest that IDC is a complex trait, likely involving multiple genetic variants, as well as environmental/comorbid condition interactions. As such, the *CACNB4* variant we identified may represent one of multiple predisposing genetic variants in African Americans. The distinct differences in clinical characteristics of IDC between African Americans and European Caucasians strongly support the inclusion of African American subjects in genetic studies of this disorder and more generally the importance of examining diverse populations to define racial differences in risk and prognoses phenotypes in IDC and other forms of chronic heart failure. We would also like to emphasize that our GWAS findings are exploratory, and a next step would be to replicate our findings in an independent cohort of African Americans. Currently, the Precision Medicine Study of Dilated Cardiomyopathy (funded by NIH in 2015) aims to phenotype and perform exome-sequencing on 1300 people with dilated cardiomyopathy (including 600 African American) and their 5200 family members from 11 sites across the United States [10]. Combining results and or performing joint analyses of all available African American data sets will be an important future direction. In addition, large cohorts with genomics data such as the USA Department of Veterans Affairs' Million Veteran Program targeting for 1 million participants [52] may be useful for this purpose too. Given the differences in cardiomyopathy rates between African Americans and European Caucasians, analytic approaches such as admixture mapping might also be helpful. Although there is biologic plausibility supporting our findings, further in vitro, human tissue, or genetically modified mouse studies of the *CACNB4* intronic region will be required to establish the mechanism by which the variation causes predisposition to IDC.

## Figures and Tables

**Table 1:** Top genome-wide association hits at *p* < 1.0 × 10^−6^.

Genes	#SNPs	CHR	SNP ID	Position	Minor Allele	Reference Allele	MAF Cases	MAF Controls	OR	*p*-Value
**(intronic)** ***CACNB4***			rs150793926	152781063	GTA	G	0.04	0.07	0.49	4.10 × 10^−8^
	8	2	rs113760736	152800750	C	T	0.03	0.07	0.50	1.50 × 10^−7^
			rs12623883	152790117	A	G	0.03	0.06	0.50	2.45 × 10^−7^
**(intronic)** ***MGAM***	1	7	rs4341082	141759846	T	C	0.38	0.47	0.70	4.50 × 10^−7^
**(intergenic)** ***THSD7A****, **THEM106B***	1	7	rs74676849	11889492	G	A	0.1	0.05	2.03	6.80 × 10^−7^

SNPs: single nucleotide polymorphisms or short indels; CHR: chromosome; OR: odds ratio.

**Table 2:** Canonical pathway analysis on the top 1000 genomic loci associated with idiopathic dilated cardiomyopathy (IDC) using the Ingenuity database. Pathways with enrichment *p* < 0.1 after corrected for multiple testing are shown here, and are grouped by tissue systems.

Classification	Canonical Pathways ^$^	Rank	*p*-Value	Corrected *p*-Value *	Genes in the Pathway That Are Associated with IDC
Cardiovascular system	Role of NFAT in cardiac hypertrophy	1	4.3 × 10^−5^	0.01	*GNAI1*, *PLCB1*, *CAMK2B*, *SRC*, *CALM1*, *PIK3R4*, *MRAS*, *CAMK1D*, *PPP3CC*, *IGF1*, *PLCG2*, *IGF1R*, *CAMK2G*, *ADCY2*, *CAMK1G*, *PLCL1*, *ADCY8*, *MEF2A*, *ADCY9*
	Cellular effects of sildenafil (Viagra)	6	3.4 × 10^−4^	0.02	*PLCB1*, *MYH7*, *CALM1*, *PRKG2*, *PRKG1*, *PLCG2*, *CACNA1C*, *ADCY2*, *PDE3A*, *ACTA1*, *KCNQ3*, *PLCL1*, *ADCY8*, *ADCY9*
	Cardiac hypertrophy signaling	17	4.4 × 10^−3^	0.10	*GNAI1*, *PLCB1*, *CALM1*, *PIK3R4*, *MRAS*, *ADRA1D*, *PPP3CC*, *IGF1*, *PLCG2*, *IGF1R*, *CACNA1C*, *ADCY2*, *ADRA2A*, *PLCL1*, *ADCY8*, *MEF2A*, *ADCY9*
Cellular signals	cAMP-mediated signaling	2	7.8 × 10^−5^	0.01	*GNAI1*, *CAMK2B*, *SRC*, *CALM1*, *CHRM3*, *CAMK1D*, *PPP3CC*, *PDE8A*, *CAMK2G*, *GRM7*, *ADCY2*, *PDE3A*, *CAMK1G*, *ADRA2A*, *OPRM1*, *ADCY8*, *AKAP6*, *ADCY9*, *AKAP1*, *HTR4*, *XCR1*
	Chemokine signaling	5	3.0 × 10^−4^	0.02	*GNAI1*, *PLCB1*, *CAMK2B*, *SRC*, *CALM1*, *MRAS*, *CAMK2G*, *CAMK1D*, *CAMK1G*, *PLCG2*
	Gap junction signaling	8	7.2 × 10^−4^	0.03	*GNAI1*, *PLCB1*, *SRC*, *PIK3R4*, *MRAS*, *PRKG2*, *PRKG1*, *PPP3CC*, *PLCG2*, *TUBB2B*, *ADCY2*, *ACTA1*, *PLCL1*, *ADCY8*, *ADCY9*
	RhoA signaling	11	2.0 × 10^−3^	0.07	*IGF1R*, *PLXNA1*, *DLC1*, *SEPT9*, *ARHGAP1*, *ANLN*, *CDC42EP3*, *ACTA1*, *ARHGAP12*, *RHPN2*, *IGF1*, *RAPGEF2*
	RhoGDI signaling	12	2.2 × 10^−3^	0.07	*GNAI1*, *CDH13*, *CDH2*, *ARHGEF3*, *SRC*, *CDH12*, *MRAS*, *CDH4*, *ARHGAP1*, *WASL*, *DLC1*, *CDH10*, *ACTA1*, *CDH8*, *ARHGAP12*
	Calcium signaling	13	2.9 × 10^−3^	0.07	*CAMK2B*, *TP63*, *MYH7*, *CALM1*, *CAMK1D*, *PPP3CC*, *NFATC3*, *GRIK1*, *CAMK2G*, *CAMK1G*, *CAMKK2*, *ACTA1*, *GRIN3A*, *MEF2A*, *CHRNA9*
	Protein kinase A signaling	14	2.9 × 10^−3^	0.07	*CAMK2B*, *GNAI1*, *CALM1*, *PPP3CC*, *DCC*, *PDE3A*, *ADCY2*, *ADCY8*, *PLCL1*, *AKAP6*, *PTPRS*, *AKAP1*, *PLCB1*, *PTPRM*, *CDC25A*, *PTPRT*, *DUSP22*, *PLCG2*, *NFATC3*, *PDE8A*, *PTPN12*, *CAMK2G*, *PTPRE*, *CDC23*, *PTPN7*, *ADCY9*
	Dopamine-DARPP32 feedback in cAMP signaling	15	3.0 × 10^−3^	0.07	*GNAI1*, *PLCB1*, *CALM1*, *PRKG2*, *PRKG1*, *PPP3CC*, *PLCG2*, *CACNA1C*, *ADCY2*, *CAMKK2*, *GRIN3A*, *ADCY8*, *PLCL1*, *ADCY9*
	G-protein coupled receptor signaling	16	3.6 × 10^−3^	0.09	*GNAI1*, *PLCB1*, *CAMK2B*, *SRC*, *PIK3R4*, *MRAS*, *CHRM3*, *ADRA1D*, *PDE8A*, *CAMK2G*, *GRM7*, *ADCY2*, *PDE3A*, *ADRA2A*, *OPRM1*, *ADCY8*, *ADCY9*, *HTR4*, *XCR1*
Neuronal/neuromuscular system	Neuropathic pain signaling in dorsal horn neurons	3	8.9 × 10^−5^	0.01	*PLCB1*, *CAMK2B*, *SRC*, *TACR1*, *PIK3R4*, *CAMK1D*, *PLCG2*, *CAMK2G*, *GRM7*, *CAMK1G*, *GRIN3A*, *KCNQ3*, *PLCL1*
	Glioma signaling	4	2.2 × 10^−4^	0.02	*IGF1R*, *CAMK2B*, *CALM1*, *MRAS*, *PIK3R4*, *CAMK2G*, *CAMK1D*, *CAMK1G*, *E2F3*, *IGF1*, *IGF2R*, *PLCG2*
	Netrin signaling	7	5.4 × 10^−4^	0.03	*NFATC3*, *UNC5C*, *ABLIM3*, *NCK2*, *PRKG1*, *DCC*, *PPP3CC*
	Synaptic long-term potentiation	9	1.7 × 10^−3^	0.07	*PLCB1*, *CAMK2B*, *CALM1*, *MRAS*, *CAMK2G*, *GRM7*, *CACNA1C*, *PPP3CC*, *PLCL1*, *ADCY8*, *GRIN3A*, *PLCG2*
	CREB signaling in neurons	10	1.9 × 10^−3^	0.07	*GNAI1*,*PLCB1*,*CAMK2B*,*CALM1*,*PIK3R4*,*MRAS*, *GRIK2*,*PLCG2*,*GRIK1*,*CAMK2G*,*GRM7*, *ADCY2*,*PLCL1*,*ADCY8*,*ADCY9*

* Benjamini–Hochberg false discovery rate corrected *p*. ^$^ Abbreviations in the pathway names: NFAT, nuclear factor of activated T cells; RhoGDI, Rho GDP-dissociation inhibitor; DARPP32, Dopamine- and cAMP-regulated phosphoprotein, Mr 32 kDa; CREB, cAMP-responsive element binding protein.

## Supplementary Materials

The following are available online at http://www.mdpi.com/2075-4426/8/1/11/s1, Table S1: Demographics of cases and controls by recruiting sites; Table S2: Top SNPs associated with IDC at *p* < 1 × 10−5; Table S3: Previous reported GWAS hits for primary cardiomyopathy; Table S4: KEGG Pathway analysis on the top 1000 genomic loci associated with IDC; Table S5: Gene-based association analysis results; Table S6: Transcriptome prediction association analysis results; Figure S1: Genotype data QC workflow; Figure S2: genetic ancestry comparison between cases and controls; Figure S3: The genetic variance accounted for by each principal component; Figure S4: Genetic ancestry of African American cases with HAPMAP 3 populations as references; Figure S5: Histogram of quality assessment parameters for imputation; Figure S6: Heritability accounted for by each chromosome; Figure S7: Quantile–quantile plots of genome-wide association *p* values; Figure S8: Regional association plot after conditioning on the top SNP rs150793926; Figure S9: Regulatory elements in *CACNB4* loci.
