# Supplementary material for: A Genome-Wide Association Study of Idiopathic Dilated Cardiomyopathy in African Americans
Source: J Pers Med. 2018 Feb 26;8(1):11. doi: 10.3390/jpm8010011 (PMC5872085; doi:10.3390/jpm8010011)

Genome wide association study of idiopathic dilated cardiomyopathy in African Americans

Huichun Xu, Gerald W. Dorn II , Amol Shetty, Ankita Parihar, Tushar Dave, Shawn W. Robinson, Stephen S. Gottlieb, Mark P. Donahue, Gordon F. Tomaselli, William E. Kraus, Braxton D. Mitchell, Stephen B. Liggett

Tables of Contents

Supplementary Table 1. Demographics of cases and controls by recruiting sites.

Supplementary Table 2. Top SNPs associated with IDC at $p < 1 \times 10^{-5}$.

Supplementary Table 3. Previous reported GWAS hits for primary cardiomyopathy**.**

Supplementary Table 4. KEGG Pathway analysis on top 1000 genomic loci associated with IDC

Supplementary Table 5. Gene-based association analysis result.

Supplementary Table 6. Transcriptome prediction association analysis result.

Supplementary Figure 1. Genotype data QC workflow.

Supplementary Figure 2. Genetic ancestry comparison between cases and controls.

Supplementary Figure 3. The genetic variance accounted by each principal component

Supplementary Figure 4: Genetic ancestry of African American cases with HAPMAP 3 populations as references.

Supplementary Figure 5. Histogram of quality assessment parameters for imputation

Supplementary Figure 6. Heritability accounted by each chromosome

Supplementary Figure 7. Quantile-quantile plots of genome wide association p values

Supplementary Figure 8. Regional association plot after conditioning on the top SNP rs150793926.

Supplementary Figure 9. Regulatory elements in *CACNB4* loci.

Supplementary Table 1. Demographics of cases and controls by recruiting sites. *P* was derived from *t*-test or chi-squared test.

	Cases (By Sites)					Cases	Controls	P (cases vs controls)
	Cincinnati	UMD	Duke	JHU	VA Commonwealth			
N (passing quality filter)	266	148	103	100	45	662	1138	
Age (Years)	57.3±13.5	52.5±13.6	51.7±12.0	52.9±12.1	56.7±11.4	54.7±12.9	62.6±10.3	<0.0001
Female(%)	47.7%	28.4%	42.7%	37.0%	33.3%	40.0%	52.9%	<0.001

Cincinnati: University of Cincinnati; UMD: University of Maryland Baltimore; JHU: Johns Hopkins Medical Institution; Duke: Duke University; VA Commonweath: Virginia Commonwealth University

Supplementary Table 2. Top SNPs associated with IDC at GWA $p < 1 \times 10^{-5}$. Post-GWAS filtering was applied: MAF > 1%, HWE $p < 1 \times 10^{-6}$, imputation quality score "info" > 0.3.

SNP	Chr	Position	Risk allele	Protective allele	categories	Nearest Genes	P-val	OR(95%CI)	Risk_alele_frequency	
									cases(%)	controls(%)
rs1846594	3	112916203	A	G	intergenic	LOC101929717,BOC	3.6E-09	1.49(1.29,1.71)	65.3	55.9
rs150793926	2	152781063	G	GTA	intronic	CACNB4	4.1E-08	1.04(1.07,28.52)	3.5	6.9
rs146580444	2	152783086	G	GA	intronic	CACNB4	1.1E-07	1.03(1.07,29.89)	3.3	6.5
rs113760736	2	152800750	C	T	intronic	CACNB4	1.5E-07	1.03(1.07,29.75)	3.4	6.7
rs74535921	2	152805237	C	T	intronic	CACNB4	2.3E-07	1.04(1.07,29.38)	3.4	6.6
rs12623883	2	152790117	A	G	intronic	CACNB4	2.5E-07	1.03(1.07,30.1)	3.3	6.5
rs16830462	2	152790808	A	G	intronic	CACNB4	2.6E-07	1.03(1.07,30.15)	3.3	6.5
rs4341082	7	141759846	C	T	intronic	MGAM	4.5E-07	1.62(1.88,2.62)	38.2	46.9
rs74676849	7	11889492	G	A	intergenic	THSD7A,TMEM106B	6.8E-07	2.03(1.58,2.62)	10.3	5.3
rs4553822	2	152777395	G	A	intronic	CACNB4	7.3E-07	1.04(1.07,26.9)	3.7	6.7
rs12618445	2	152806958	A	G	intronic	CACNB4	8.7E-07	1.03(1.07,30.09)	3.3	6.3
rs6556795	5	164467492	G	A	intergenic	LOC102546299,CTB-7E3.1	1.2E-06	1.36(1.18,1.57)	67.3	60.2
rs7486169	12	74732024	G	A	intergenic	LOC100507377,ATXN7L3B	1.5E-06	1.07(1.12,15.22)	6.6	10.8
rs202209255	13	106369082	TC	T	ncRNA_intronic	LINC00343	1.7E-06	1.81(1.32,2.48)	6.3	3.6
(NA)	10	45742058	G	T	intergenic	ANKRD30BP3,OR13A1	1.8E-06	1.24(1.36,5.23)	19.1	26.6
rs145200041	5	164470241	G	T	intergenic	LOC102546299,CTB-7E3.1	1.8E-06	1.39(1.2,1.61)	70.1	62.7
rs77698332	11	36872764	G	A	intergenic	C11orf74,LOC103312105	2.5E-06	2.12(1.56,2.88)	7.1	3.5
rs72654150	4	88523577	T	C	intergenic	SPARCL1,DSPP	2.5E-06	1.03(1.05,40.45)	2.5	4.8
rs11765910	7	131565550	T	C	intergenic	PODXL,LOC101928782	2.5E-06	1.28(1.06,1.55)	15.5	12.6
rs7627580	3	56797768	G	T	intronic	ARHGEF3	2.6E-06	1.39(1.21,1.61)	69.6	62.2
rs7737487	5	164483002	A	G	intergenic	LOC102546299,CTB-7E3.1	2.7E-06	1.36(1.18,1.57)	68.7	61.8
rs35548982	3	151389203	GT	G	ncRNA_intronic	MIR548H2	3.1E-06	1.96(1.45,2.66)	95.7	91.8
rs11862795	16	1327392	C	T	intergenic	TPSD1,UBE2I	3.1E-06	1.84(1.36,2.49)	6.8	3.8
rs4701446	5	24478826	G	A	intergenic	PRDM9,CDH10	3.2E-06	1.2(1.3,5.98)	16.7	23.1
rs1578205	10	45748743	C	T	intergenic	ANKRD30BP3,OR13A1	3.3E-06	1.21(1.32,5.87)	17.0	24.2
rs4948978	10	45745850	T	A	intergenic	ANKRD30BP3,OR13A1	3.5E-06	1.21(1.32,5.86)	17.1	24.2
rs115624974	17	57904302	A	C	intronic	VMP1	3.6E-06	1.01(1.03,90.9)	1.1	2.8
rs138238284	17	57871006	G	T	intronic	VMP1	3.7E-06	1.01(1.03,90.78)	1.1	2.8
rs142971575	3	192179346	CTTAAT	C	intronic	FGF12	4.3E-06	1.05(1.08,20.22)	4.9	7.4
rs17016480	1	206989111	C	A	intronic	IL19	4.3E-06	1.44(1.54,3.28)	30.5	35.0
(NA)	3	151404254	A	T	ncRNA_intronic	MIR548H2	4.4E-06	2.11(1.51,2.94)	96.5	92.8
rs73464515	7	143634271	C	T	downstream	OR2F2	4.6E-06	2.42(1.62,3.6)	4.4	1.9
rs113620126	7	143635254	T	C	intergenic	OR2F2,OR2F1	4.6E-06	2.42(1.62,3.6)	4.4	1.9
rs60792095	7	143612396	A	G	intergenic	FAM115A,OR2F2	4.6E-06	2.41(1.62,3.59)	4.4	1.9
rs73462438	7	143619047	G	A	intergenic	FAM115A,OR2F2	4.6E-06	2.41(1.62,3.6)	4.4	1.9
(NA)	10	45744479	C	T	intergenic	ANKRD30BP3,OR13A1	4.6E-06	1.21(1.32,5.83)	17.1	24.2
rs17052317	8	24517224	G	A	intergenic	LOC101929294,NEFM	4.8E-06	1.94(1.44,2.62)	7.2	3.8

rs4487073	2	205246706	C	T	intergenic	ICOS,PARD3B	4.8E-06	2.03(2.31,1.97)	50.6	56.8
rs11742119	5	88780645	T	C	intergenic	MEF2C-AS1,MIR3660	4.9E-06	1.02(1.04,65.16)	1.5	4.1
rs60994302	8	24496566	G	A	intergenic	LOC101929294,NEFM	5.0E-06	1.98(1.45,2.7)	6.7	3.5
rs73554942	8	24497512	C	T	intergenic	LOC101929294,NEFM	5.0E-06	1.98(1.45,2.7)	6.7	3.5
rs73464595	7	143665764	A	G	intergenic	OR2F1,OR6B1	5.0E-06	2.4(1.61,3.59)	4.4	1.9
rs60837928	8	24504737	A	G	intergenic	LOC101929294,NEFM	5.4E-06	1.98(1.46,2.67)	7.0	3.7
rs2649734	3	157347640	A	G	intergenic	PQLC2L,SHOX2	5.5E-06	1.41(1.22,1.62)	68.8	61.1
rs10272945	7	9606276	C	G	intergenic	NXPH1,PER4	5.5E-06	1.05(1.11,19.63)	5.1	10.0
rs4426938	5	164481641	A	T	intergenic	LOC102546299,CTB-7E3.1	5.6E-06	1.32(1.15,1.52)	65.1	58.5
(NA)	7	141759994	T	C	intronic	MGAM	5.6E-06	1.39(1.21,1.59)	61.7	53.8
rs78212518	17	76556115	T	C	intronic	DNAH17	5.7E-06	2.83(1.8,4.46)	3.7	1.4
rs11182052	12	38450421	T	C	intergenic	NONE,ALG10B	5.8E-06	1.33(1.48,3.99)	25.1	32.4
rs10503754	8	24515323	C	T	intergenic	LOC101929294,NEFM	5.9E-06	1.87(1.4,2.51)	7.5	4.1
rs17052298	8	24506696	G	A	intergenic	LOC101929294,NEFM	6.0E-06	1.96(1.44,2.68)	6.7	3.5
rs202107652	12	38450336	C	CT	intergenic	NONE,ALG10B	6.1E-06	1.1(1.13,11.24)	8.9	11.6
rs10879773	12	74819080	G	A	intergenic	LOC100507377,ATXN7L3B	6.5E-06	1.07(1.12,15.01)	6.7	10.6
rs149932627	12	114861677	C	T	intergenic	TBX5-AS1,TBX3	6.5E-06	2.55(1.56,4.19)	3.0	1.2
rs2244757	4	24887391	T	C	intronic	CCDC149	6.6E-06	15.04(26.82,1.07)	93.3	96.3
rs149215314	6	170701831	C	G	intronic	FAM120B	6.6E-06	2.09(1.44,3.02)	4.8	2.4
rs78580051	16	1327197	A	G	intergenic	TPSD1,UBE2I	6.7E-06	1.8(1.34,2.42)	7.1	4.1
rs114241858	20	46949149	C	G	intergenic	LINC01522,LINC00494	6.8E-06	1.71(1.32,2.22)	9.0	5.5
rs140035275	20	32652803	T	C	intronic	RALY	6.9E-06	2.44(1.59,3.75)	3.9	1.6
rs146955135	2	198558368	C	T	intergenic	RFTN2,MARS2	6.9E-06	1.03(1.07,32.79)	3.0	6.1
rs75330306	2	198545864	A	G	intergenic	RFTN2,MARS2	6.9E-06	1.03(1.07,32.78)	3.1	6.1
rs78779226	5	88794355	A	G	intergenic	MEF2C-AS1,MIR3660	7.0E-06	1.02(1.04,63.01)	1.6	4.1
rs146317878	4	88537357	T	C	exonic	DSPP	7.0E-06	1.03(1.05,39.5)	2.5	4.8
rs9764469	5	164469246	G	A	intergenic	LOC102546299,CTB-7E3.1	7.0E-06	1.35(1.17,1.56)	68.6	61.8
(NA)	7	92177567	AT	A	intergenic	RBM48,FAM133B	7.3E-06	1.74(1.37,2.19)	11.4	6.9
rs112019249	7	11884071	A	G	intergenic	THSD7A,TMEM106B	7.4E-06	1.69(1.39,2.05)	17.5	11.2
rs145355401	11	69391962	C	T	intergenic	LINC01488,CCND1	7.4E-06	2.48(1.67,3.68)	4.6	1.9
rs150368909	16	1327118	C	CA	intergenic	TPSD1,UBE2I	7.5E-06	1.79(1.33,2.4)	7.1	4.1
rs8047442	16	78764097	C	T	intronic	WWOX	7.7E-06	1.34(1.17,1.54)	43.8	36.8
rs142306890	20	32661802	T	C	intronic	RALY	8.1E-06	2.43(1.57,3.74)	3.8	1.6
rs8051448	16	87678441	C	T	exonic	JPH3	8.4E-06	1.14(1.2,7.98)	12.5	16.9
rs57006319	2	152758611	C	T	intronic	CACNB4	8.5E-06	1.03(1.06,35.58)	2.8	5.3
rs140644323	6	148587236	G	A	intergenic	SAMD5,SASH1	8.7E-06	1.01(1.03,74.47)	1.3	3.2
rs73308970	20	61773457	A	G	intergenic	HAR1A,MIR124-3	8.8E-06	1.06(1.09,18.94)	5.3	8.2
rs113241821	7	11890459	A	C	intergenic	THSD7A,TMEM106B	9.0E-06	1.69(1.39,2.04)	17.5	11.2
rs77283538	8	24520575	T	C	intergenic	LOC101929294,NEFM	9.0E-06	1.89(1.4,2.57)	6.8	3.7
rs7807617	7	11890992	A	G	intergenic	THSD7A,TMEM106B	9.0E-06	1.69(1.39,2.04)	17.5	11.2
rs16867696	5	88794820	A	C	intergenic	MEF2C-AS1,MIR3660	9.1E-06	1.02(1.05,57.5)	1.7	4.3
rs61815623	1	194211195	T	G	intergenic	LINC01031,KCNT2	9.2E-06	1.74(1.25,2.44)	5.4	3.2

rs192183165	6	12928850	T	C	intronic	PHACTR1	9.2E-06	1.44(1.25,1.66)	38.3	30.1
rs61815639	1	194240495	T	C	intergenic	LINC01031,KCNT2	9.2E-06	1.76(1.26,2.45)	5.5	3.2
rs112121754	6	12928854	G	T	intronic	PHACTR1	9.3E-06	1.44(1.25,1.66)	38.3	30.1
rs59814330	8	24523465	T	C	intergenic	LOC101929294,NEFM	9.4E-06	1.8(1.34,2.41)	7.2	4.1
rs139236944	2	198646026	G	A	intronic	BOLL	9.4E-06	1.03(1.07,32.64)	3.1	6.1
rs114108584	4	177821987	T	C	intergenic	VEGFC,NEIL3	9.4E-06	1.03(1.06,35.71)	2.8	5.5
rs28394614	7	9602342	A	G	intergenic	NXPH1,PER4	9.5E-06	1.05(1.11,20.38)	4.9	9.6
rs186593276	20	32716973	A	G	intergenic	EIF2S2,ASIP	9.5E-06	2.45(1.59,3.77)	3.8	1.6
rs67927590	7	122693767	A	G	intergenic	TAS2R16,SLC13A1	9.7E-06	1.42(1.58,3.37)	29.7	36.6
rs2264512	11	79425060	G	A	intergenic	TENM4,LOC101928944	9.7E-06	1.27(1.37,4.72)	21.2	27.0
rs7959611	12	74583330	T	C	ncRNA_intronic	LOC100507377	9.9E-06	1.61(1.28,2.03)	91.6	87.1
rs61742179	17	76556982	C	T	exonic	DNAH17	9.9E-06	2.45(1.66,3.6)	4.8	2.0
rs75611182	5	164470168	G	A	intergenic	LOC102546299,CTB-7E3.1	1.0E-05	1.32(1.15,1.52)	62.0	55.2

Supplementary Table 3. Previous reported GWAS hits for primary cardiomyopathy.

PUBMED_ID	Disease	SNPs	Risk-Allele	Context	Genes	OR(reported)	OR(Current study)	P-val(current study)
20975947[1]	IDC	rs1739843	C	intronic	*HSPB7*	1.39[1.54-1.28]	1.32(1.13,1.54)	0.0048*
23853074[2]	IDC	rs9262636	G	intron	*HCG22*	1.20[1.11–1.28]	0.82(0.68,0.97)	0.091
23853074[2]	IDC	rs9262635	G	intron	*HCG23*	1.48[1.29–1.68]	0.82(0.68,0.97)	0.080
21459883[3]	IDC	rs10927875	C	intron	*ZBTB17*	1.32[1.19-1.43]	1.22(1.44,1.03)	0.166
21459883[3]	IDC	rs2234962	T	missense	*BAG3*	1.52[1.22-1.89]	1.44(2.04,1.01)	0.172
23255317[4]	HCM	rs516514	T	intron	*FHOD3*	2.45[1.76–3.41]	1.05(0.92,1.20)	0.617
23255317[4]	HCM	rs2303510	A	intron	*FHOD3*	2.01[1.64–2.64]	0.94(0.81,1.08)	0.803

IDC: Idiopathic dilated cardiomyopathy ; HCM: Hypertrophic cardiomyopathy.

References

1. Stark K, Esslinger UB, Reinhard W, Petrov G, Winkler T, Komajda M, Isnard R, Charron P, Villard E, Cambien F, et al: **Genetic association study identifies HSPB7 as a risk gene for idiopathic dilated cardiomyopathy.** *PLoS Genet* 2010, **6:**e1001167.
2. Meder B, Ruhle F, Weis T, Homuth G, Keller A, Franke J, Peil B, Lorenzo Bermejo J, Frese K, Huge A, et al: **A genome-wide association study identifies 6p21 as novel risk locus for dilated cardiomyopathy.** *Eur Heart J* 2014, **35:**1069-1077.
3. Villard E, Perret C, Gary F, Proust C, Dilanian G, Hengstenberg C, Ruppert V, Arbustini E, Wichter T, Germain M, et al: **A genome-wide association study identifies two loci associated with heart failure due to dilated cardiomyopathy.** *Eur Heart J* 2011, **32:**1065-1076.
4. Wooten EC, Hebl VB, Wolf MJ, Greytak SR, Orr NM, Draper I, Calvino JE, Kapur NK, Maron MS, Kullo IJ, et al: **Formin homology 2 domain containing 3 variants associated with hypertrophic cardiomyopathy.** *Circ Cardiovasc Genet* 2013, **6:**10-18.

Supplementary Table 4. KEGG Pathway analysis on top 1000 genomic loci associated with IDC. Pathways were clustered based on the overlapping of their member genes. Only those clusters with at least one pathway *p*-value < 0.05 were included.

Term	PValue	Count	Genes
**Annotation Cluster 1**	**Enrichment Score: 1.79**		
hsa04020:Calcium signaling pathway	0.002	17	ADCY2, TACR2, ADCY8, CAMK2G, TACR1, HTR4, ITPKB, NTSR1, ADCY9, CHRM3, PLCG2, CALM3, PPP3CC, CAMK2B, PLCB1, CACNA1C, ADRA1D
hsa04912:GnRH signaling pathway	0.019	10	ADCY2, ADCY9, ADCY8, CAMK2G, CALM3, PLA2G6, CAMK2B, PLCB1, CACNA1C, SRC
hsa04720:Long-term potentiation	0.021	8	ADCY8, RPS6KA2, CAMK2G, CALM3, PPP3CC, CAMK2B, PLCB1, CACNA1C
hsa04540:Gap junction	0.030	9	ADCY2, TUBB2B, ADCY9, GNAI1, ADCY8, PRKG2, PRKG1, PLCB1, SRC
hsa04270:Vascular smooth muscle contraction	0.091	9	ADCY2, ADCY9, ADCY8, CALM3, PLA2G6, PRKG1, PLCB1, CACNA1C, ADRA1D
**Annotation Cluster 2**	**Enrichment Score: 1.31**		
hsa05414:Dilated cardiomyopathy	0.004	11	ITGA9, ADCY2, ADCY9, ADCY8, ITGB7, IGF1, SGCD, MYH7, CACNB4, CACNA2D3, CACNA1C
hsa05412:Arrhythmogenic right ventricular cardiomyopathy (ARVC)	0.036	8	ITGA9, ITGB7, SGCD, CACNB4, CDH2, CACNA2D3, CACNA1C, CTNNA2
hsa05410:Hypertrophic cardiomyopathy	0.060	8	ITGA9, ITGB7, IGF1, SGCD, MYH7, CACNB4, CACNA2D3, CACNA1C
hsa04260:Cardiac muscle contraction	0.632	4	MYH7, CACNB4, CACNA2D3, CACNA1C
**Annotation Cluster 3**	**Enrichment Score: 0.58**		
hsa05214:Glioma	0.044	7	IGF1R, E2F3, CAMK2G, PLCG2, CALM3, IGF1, CAMK2B
hsa05218:Melanoma	0.567	4	IGF1R, E2F3, IGF1, FGF12
hsa05215:Prostate cancer	0.719	4	IGF1R, E2F3, IGF1, SRD5A2

Supplementary Table 5. Top 20 genes from Gene-based association analysis using fastBAT. A gene region is defined as 50kb up- or down-stream of the boundary of a gene. LD pruning was used to remove SNPs whose LD *r*-squared values are larger than 0.9 with included SNPs.

Gene	Chr	Start	End	No.SNPs	Gene.Pvalue	TopSNP.Pval	TopSNP
CSPG5	3	47603727	47621730	123	6.7E-05	1.5E-05	rs6805353
MAP4	3	47892179	48130769	188	6.8E-05	3.5E-05	rs4858860
SMARCC1	3	47627377	47823405	190	7.0E-05	1.5E-05	rs6805353
DHX30	3	47844398	47891686	101	1.0E-04	2.7E-05	rs7641111
MIR1226	3	47891044	47891119	75	1.0E-04	3.5E-05	rs4858860
HRNR	1	152184551	1.52E+08	222	1.5E-04	2.0E-05	rs10788822
CCBE1	18	57098170	57364644	1240	1.7E-04	8.5E-05	rs9748813
RPS28	19	8386383	8387280	215	2.4E-04	1.1E-04	rs2913949
KANK3	19	8387467	8408146	234	2.9E-04	1.1E-04	rs2913949
LINC00152	2	87754973	87821030	191	3.0E-04	1.9E-04	rs12472419
ARHGAP12	10	32094325	32217804	355	3.2E-04	2.2E-05	rs2808090
NFATC3	16	68119268	68263162	204	3.3E-04	9.3E-05	rs7189381
PPIAL4B	1	149553002	1.5E+08	28	3.5E-04	3.0E-03	rs189309436
PPIAL4C	1	149553002	1.5E+08	28	3.5E-04	3.0E-03	rs189309436
MIR6773	16	68267328	68267402	120	3.7E-04	9.3E-05	rs7189381
TRPC2	11	3647689	3658789	638	3.8E-04	1.3E-04	rs10767372
ESRP2	16	68262449	68270136	126	4.6E-04	9.3E-05	rs7189381
C21orf15	21	15215453	15220685	441	4.8E-04	1.4E-05	rs117735178
MDC1	6	30667583	30685458	207	4.9E-04	6.7E-04	rs114530382
RPTN	1	152126070	1.52E+08	161	5.1E-04	2.0E-05	rs10788822

Supplementary Table 6. Top 20 genes from transcriptome wide association analysis using PrediXcan. Gene expression imputation was based on eQTL model build for heart left ventricle tissue.

Gene	Beta	SE(Beta)	p-value	Gene_Description
PRMT7	-0.86	0.23	1.3E-04	protein arginine methyltransferase 7
VIP	-0.71	0.20	3.1E-04	vasoactive intestinal peptide
ZDHHC5	-0.86	0.25	4.8E-04	zinc finger DHHC-type containing 5
KIF5B	1.10	0.31	4.8E-04	kinesin family member 5B
CCHCR1	-3.56	1.04	6.1E-04	coiled-coil alpha-helical rod protein 1
CXCL5	0.98	0.29	6.4E-04	C-X-C motif chemokine ligand 5
ATR	-0.53	0.16	8.0E-04	ATR serine/threonine kinase
AK9	-1.05	0.31	8.2E-04	adenylate kinase 9
DDX31	2.64	0.79	8.6E-04	DEAD-box helicase 31
ZNF23	-1.27	0.38	9.1E-04	zinc finger protein 23
MRPS10	0.42	0.13	1.1E-03	mitochondrial ribosomal protein S10
MUC1	-15.05	4.62	1.1E-03	mucin 1, cell surface associated
MITD1	-0.62	0.19	1.3E-03	microtubule interacting and trafficking domain containing 1
hla-dqa1	0.29	0.09	1.3E-03	major histocompatibility complex, class II, DQ alpha 1
SHPRH	-1.32	0.41	1.3E-03	SNF2 histone linker PHD RING helicase
BTBD9	1.07	0.34	1.6E-03	BTB domain containing 9
GATA3	0.31	0.10	1.8E-03	GATA binding protein 3
TOMM40	-0.90	0.29	1.9E-03	translocase of outer mitochondrial membrane 40
PTPN23	-0.61	0.19	1.9E-03	protein tyrosine phosphatase, non-receptor type 23
NME6	0.73	0.24	1.9E-03	NME/NM23 nucleoside diphosphate kinase 6

Supplementary Figure 1. Quality control analysis and filtering on directly assayed genotype data.

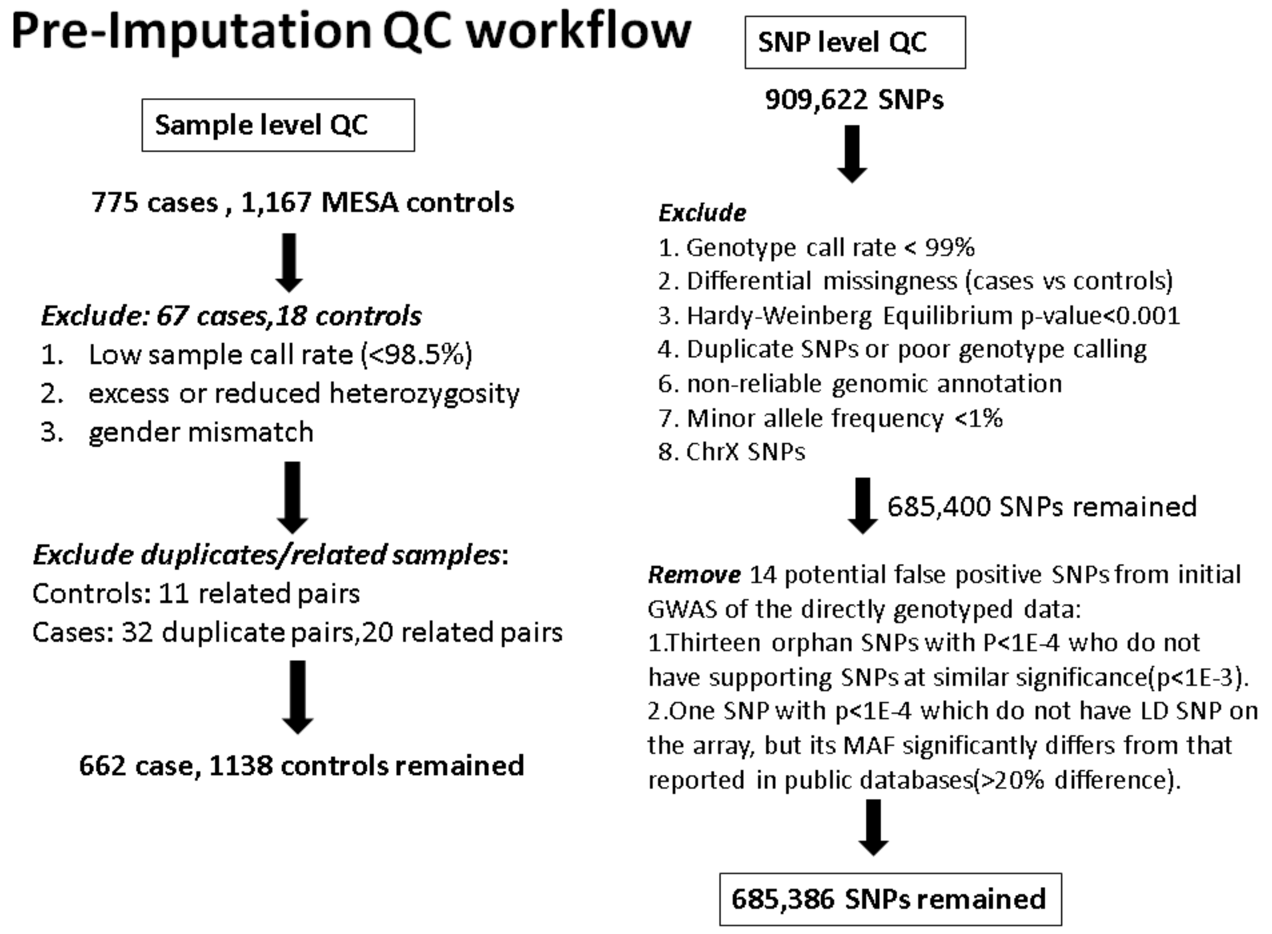

Supplementary Figure 2. Population genetic ancestry comparison between cases and controls. Principal components were calculated based on common SNPs on autosomal chromosomes after LD pruning.

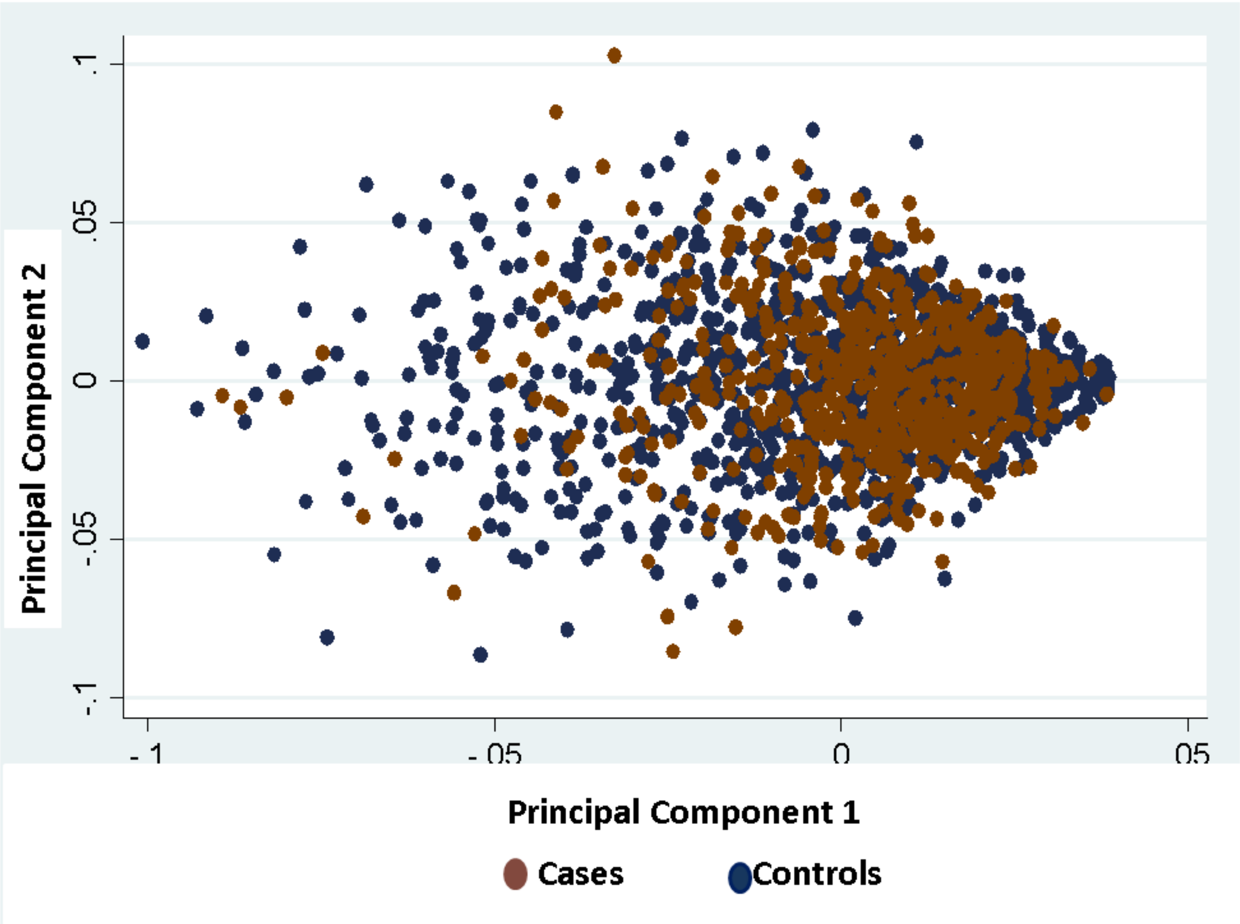

Supplementary Figure 3. The genetic variance accounted by each principal component (PC) based on analysis using Eigensoft. PC vector 1 accounted the largest amount of compared to the remaining PC vectors.

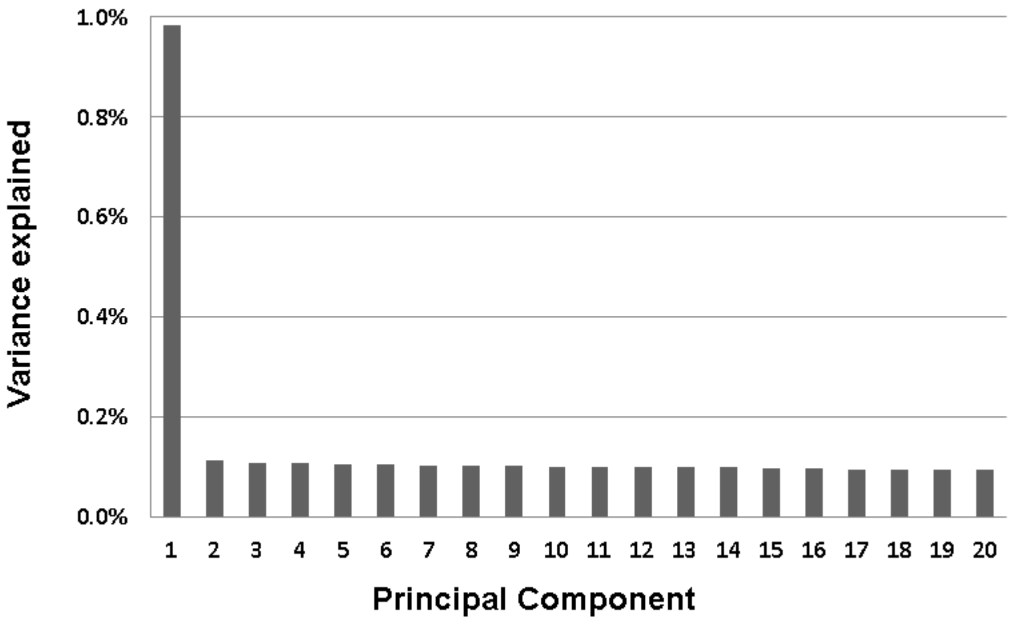

Supplementary Figure 4: Genetic ancestry of African American cases with HAPMAP 3 populations as references using principal component analysis.

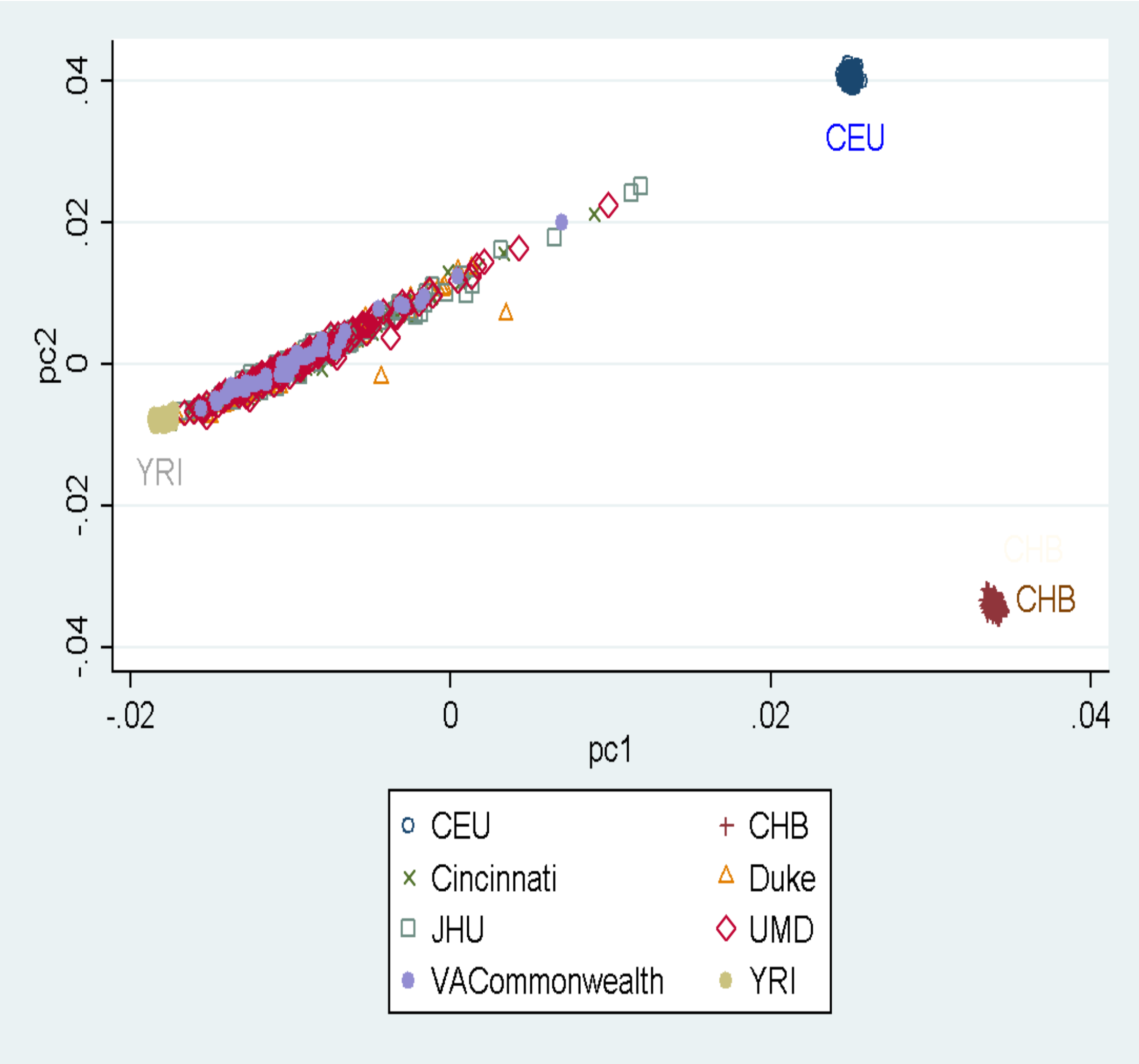

(Study sites) Cincinnati: University of Cincinnati; UMD: University of Maryland Baltimore; JHU: Johns Hopkins Medical Institution; Duke: Duke University; VA Commonweath: Virginia Commonwealth University.

Supplementary Figure 5. Histogram of quality assessment parameters for imputation. (1). Histogram of imputation quality score "info" for imputed SNPs; (2). Histogram of concordance for directly genotyped SNPs. These SNPs were taken out one at a time and imputed back. The imputed-back genotypes were then compared with original genotypes called from array data.

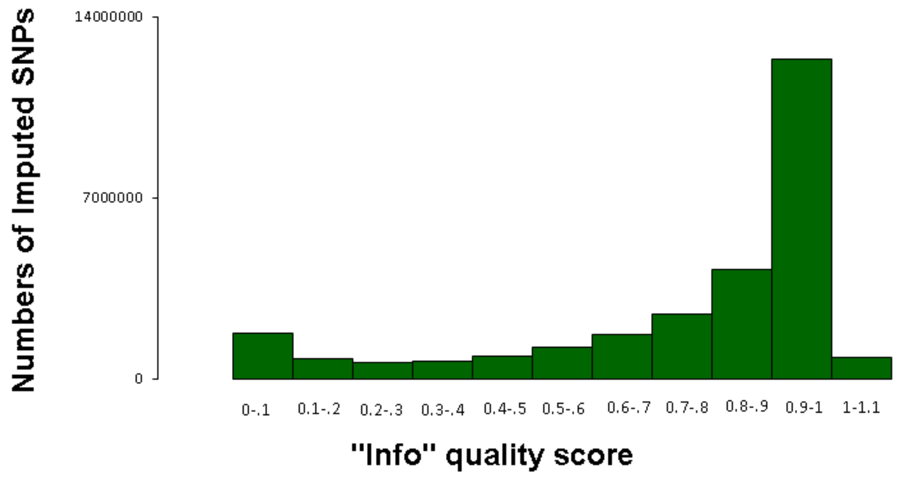

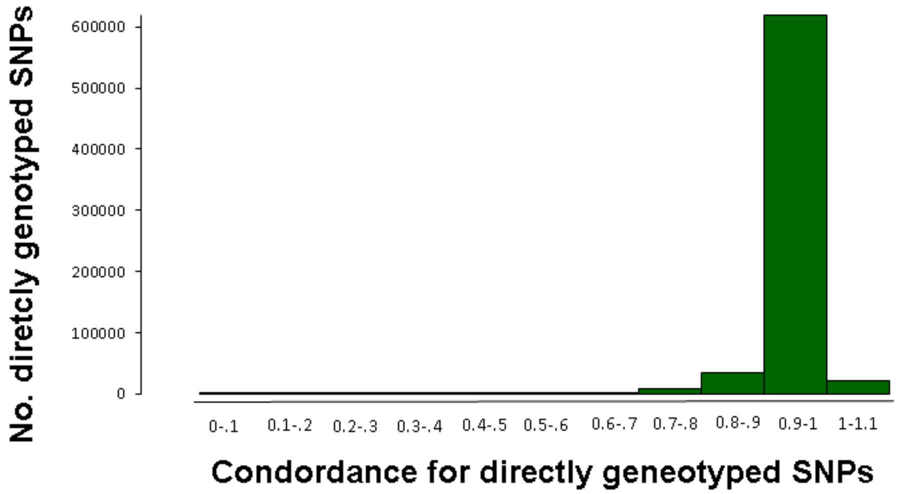

Supplementary Figure 6. Heritability analysis of each chromosome based on directly genotyped data on autosomal chromosomes.

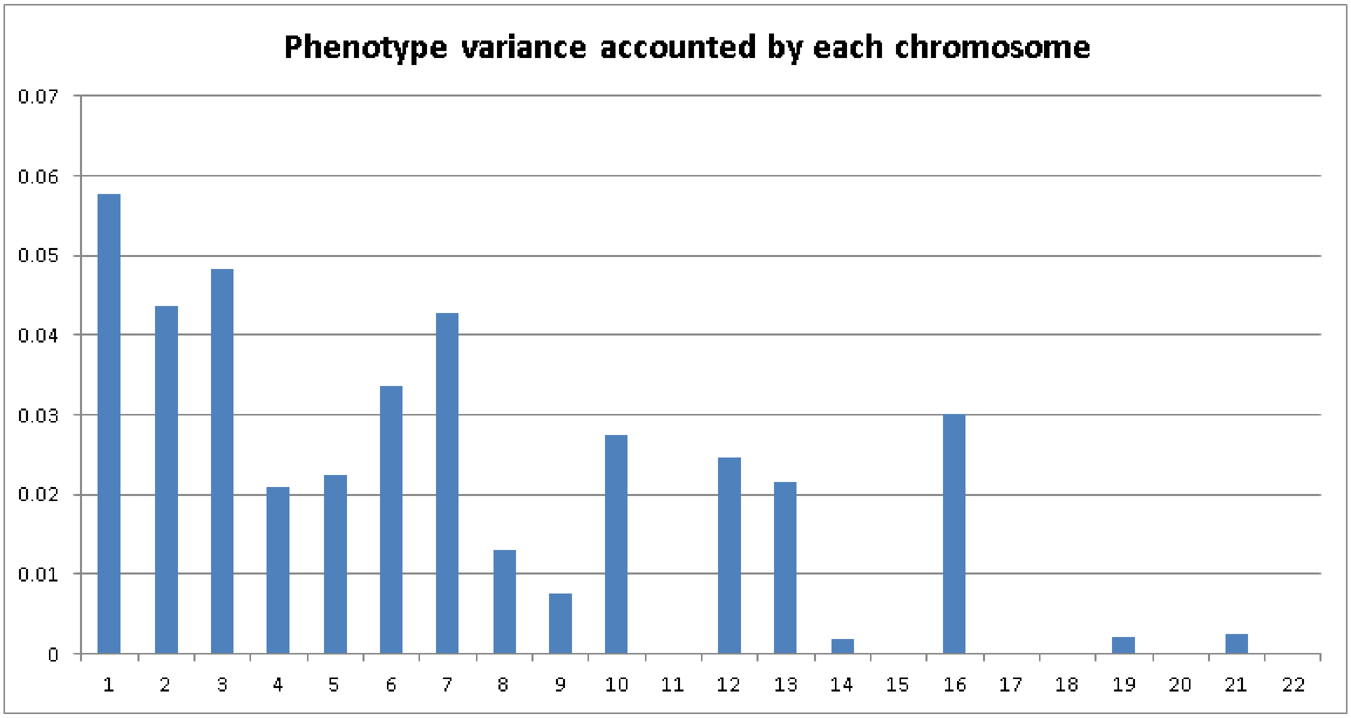

Supplementary Figure 7. Quantile-quantile plots to assess whether the obtained genome wide association *p*-values have a distribution which is conformed to what can be expected. Top panel: without post-GWAS filtering; Bottom panel: with post-GWAS filtering using cut off MAF > 1%, *p* (HWE) > 1 × 10^{-6}, and imputation quality measure "info" > 0.3.

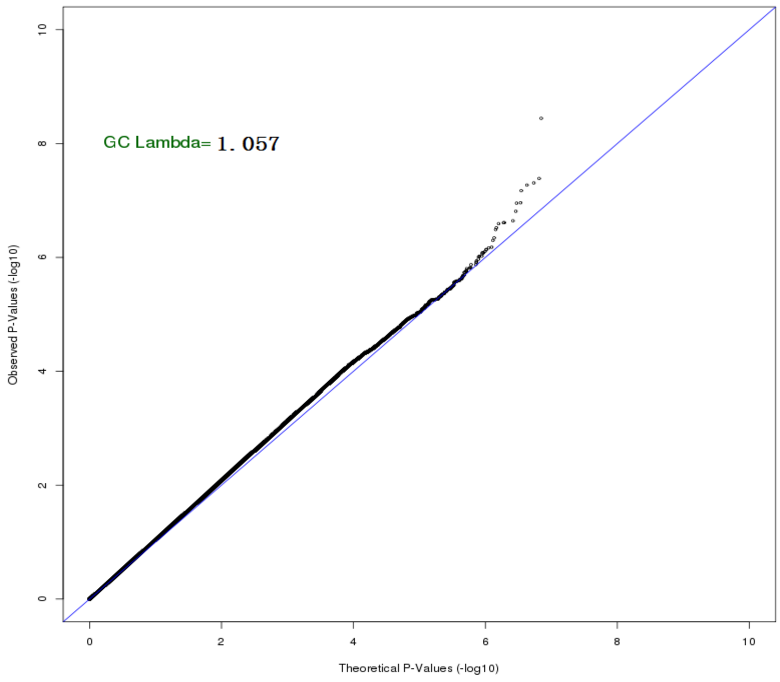

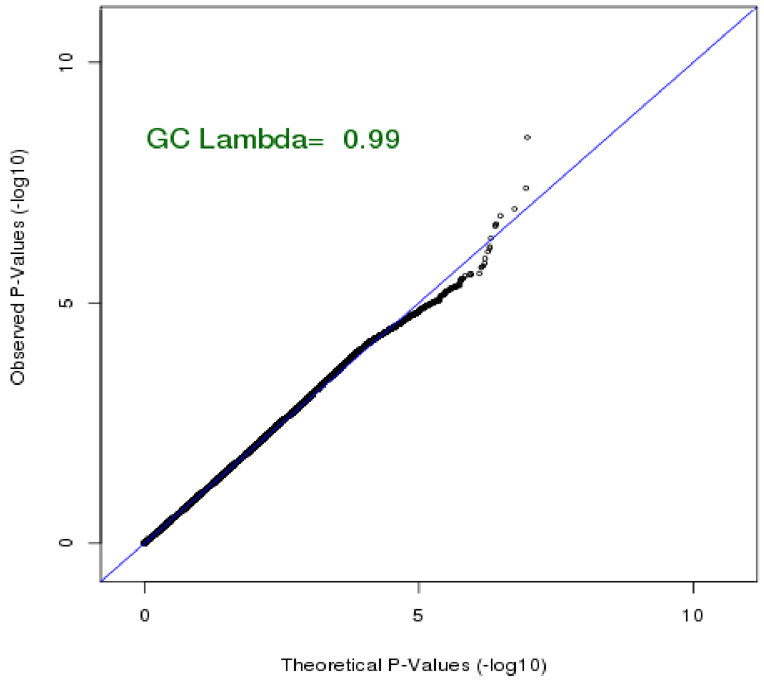

Supplementary Figure 8. Regional association plot after conditioning on the top SNP rs150793926 for the novel CACNB4 loci. Genomic coordinates were shown in *X*-axis, and –log (*p*-value) was shown in *Y*-axis to the left. Recombination rates were estimated based on 1000 Genomes Project AFR data and were shown on the *Y*-axis to the right. The index SNP is shown as purple. The r^2 values of the remaining SNPs with the index SNP are color coded as indicated by the color bar to the upper right: red being high and green being medium, light blue being low, and grey indicating no LD data available. The genes in this region are indicated at the bottom with arrows being its 5'-3' directions.

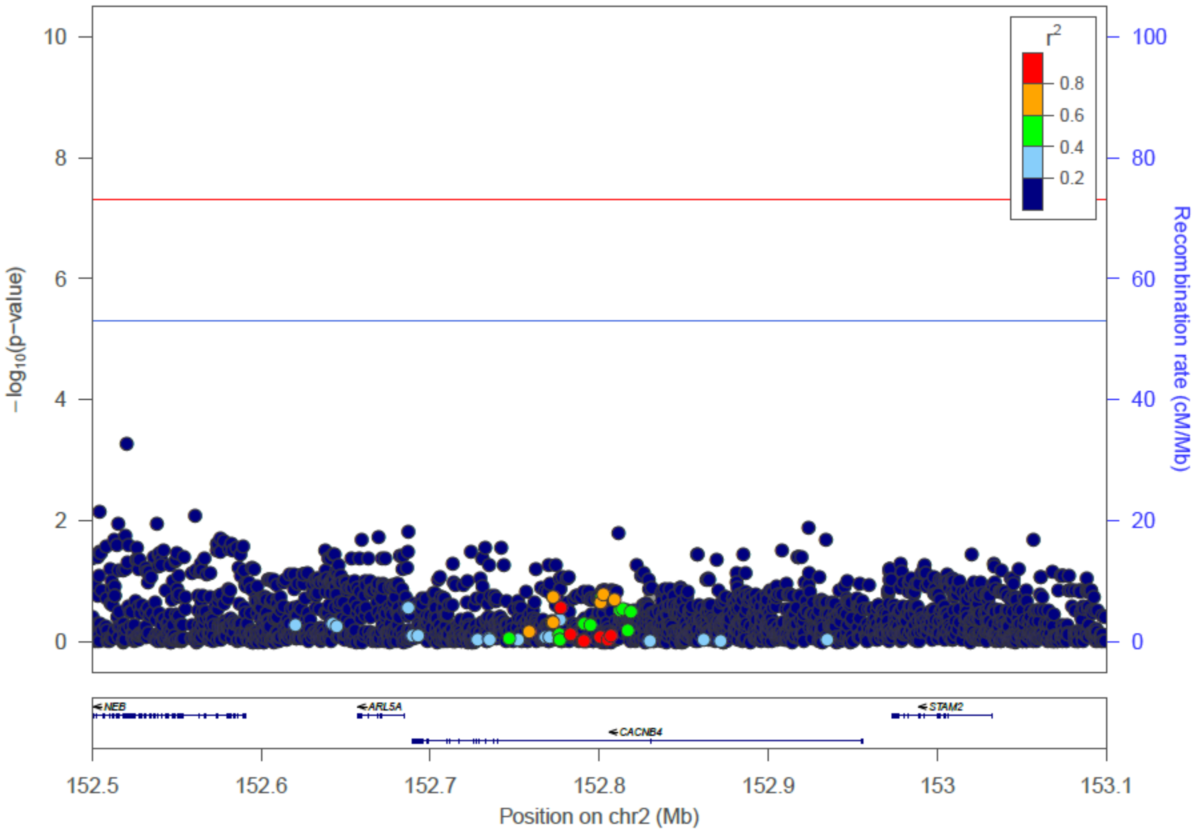

Supplementary Figure 9. Regulatory elements in our top loci in *CACNB4* gene. The boundary of this region was defined by the far left SNP and right SNP which has association p values less than 1.0 × 10^{-5}.

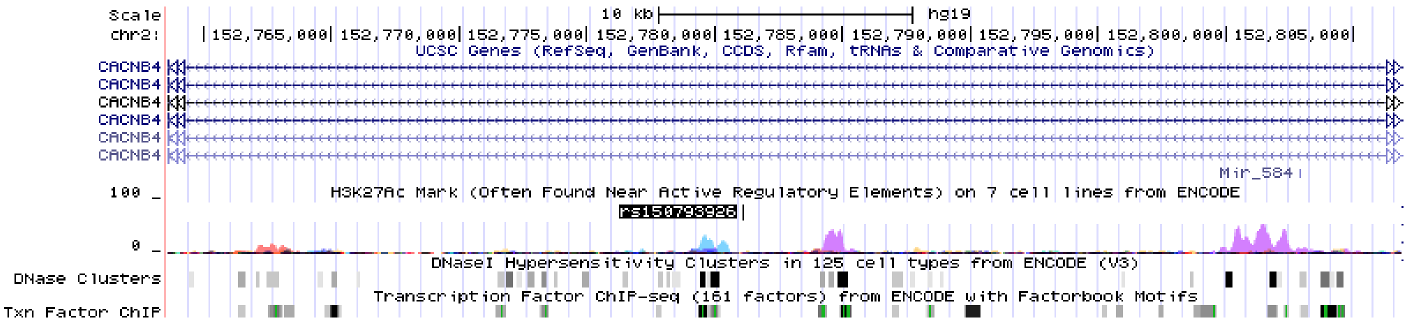

Supplement: Supplementary file 1 [file jpm-08-00011-s001.pdf]
